# Tonal Surprisal and Contextual Shifts Evoke Distinct Pupil **Dilation** During Dynamic Sound Sequences

**DOI:** 10.1111/ejn.70380

**Published:** 2026-01-04

**Authors:** Jorie J. G. van Haren, Jan‐Luca Schröder, Floris P. de Lange, Sonja A. Kotz, Federico De Martino

**Affiliations:** ^1^ Faculty of Psychology and Neuroscience, Department of Cognitive Neuroscience Maastricht University Maastricht the Netherlands; ^2^ Donders Institute for Brain, Cognition, and Behaviour Radboud University Nijmegen the Netherlands; ^3^ Transdisciplinary Research Area Life and Health, Center for Artificial Intelligence and Neuroscience University of Bonn Bonn Germany

**Keywords:** auditory sequences, Bayesian inference, locus coeruleus–norepinephrine system, predictive processing, pupillometry, statistical learning, uncertainty

## Abstract

The human brain continuously forms predictions about the unfolding sensory environment, relying on contextual information to anticipate upcoming events while remaining sensitive to unexpected changes. This study examined how pupil‐linked phasic arousal, a putative proxy for the locus coeruleus–norepinephrine system, reflects the interplay between tonal surprisal (unexpectedness) and precision (reliability of the inferred context) in dynamic auditory contexts. Twenty‐eight participants passively listened to stochastic tone sequences transitioning between periods of low‐entropy (informative context) and high‐entropy (less informative context). We quantified tone‐by‐tone surprisal and precision using Bayesian modeling. Despite their slow time evolution, pupil dilation responses revealed sensitivity to both surprisal and precision, showing that arousal tracks momentary deviations and the stability of contextual predictions. Analyses of context boundaries showed that transitions between distinct low‐entropy environments (LE‐dLE) evoked significant pupil dilation, whereas shifts between low‐ and high‐entropy environments (LE‐HE and HE‐LE) did not. These findings indicate that pupil‐linked arousal primarily responds to salient contextual shifts involving stable environments rather than to changes in entropy per se. The results emphasize the role of the locus coeruleus–norepinephrine system in adaptive model updating during passive listening and demonstrate the brain's continuous and implicit monitoring of uncertainty to navigate dynamic auditory environments.

## Graphical abstract

- Using pupil dilation and Bayesian modeling, we show that listeners track both momentary unexpectedness and the reliability of auditory contexts.
- Model‐based analyses reveal that tone‐by‐tone surprisal and contextual precision jointly shape arousal across dynamic auditory environments.
- Notably, pupil responses increased following transitions between stable, low‐entropy contexts, but not during shifts involving high‐entropy states.

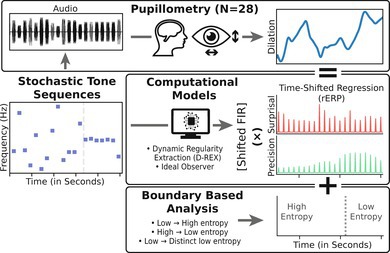

**Abbreviations**
dLE: distinct low entropy
D‐REX: Dynamic Regularity Extraction
ERCPN: Ethics Review Committee for Psychology and Neuroscience
FIR: finite impulse response
FWE: family‐wise error
GMM: Gaussian mixture model
HE: high entropy
LC: locus coeruleus
LE: low entropy
NE: norepinephrine
OSF: Open Science Framework
PDR: pupil dilation response
rERP: regression‐based event‐related potential
TFCE: threshold‐free cluster enhancement
TRF: temporal response function

## 1 Introduction

When listening, our brains continuously build an internal model of the sensory environment. This process relies on identifying and leveraging regularities in the surrounding world to form expectations about what is likely to occur next and allows the brain to efficiently process incoming information while allocating additional cognitive resources to unexpected (surprising) events (Denham and Winkler 2020; Friston 2005; Heilbron and Chait 2017). This dynamic interplay between prediction and surprisal forms the foundation of the broader framework of predictive processing. Within this framework, errors elicited by stimuli that deviate from current expectations serve as signals for the brain to refine its internal model, aligning it with the altered sensory landscape.

Predictive processes in the brain operate across multiple timescales, reflecting the complexity of navigating dynamic environments. Surprising sensory events—whether brief outliers or signs of broader contextual shifts—trigger prediction errors that disrupt ongoing predictions. Initially, the brain must balance responsiveness to transient violations with sensitivity to more persistent changes, ensuring flexibility without prematurely updating its internal model. When strong violations occur, they prompt a reevaluation of existing predictions, guiding the system to decide whether to retain, adjust, or abandon the current model of expectations (Dayan and Yu 2006; O'Reilly 2013; Sara and Bouret 2012).

A critical mediator of this adaptive process is the locus coeruleus–noropinephrine (LC‐NE) system. The LC‐NE system, located in the brainstem, is considered to play a vital role in signaling saliency of changes in the environment (Bouret and Sara 2005; Dayan and Yu 2006; Marshall 2016; Payzan‐LeNestour et al. 2013; Yu and Dayan 2005). It achieves this by releasing norepinephrine (NE), a neuromodulator that projects broadly across the brain, increasing attention and arousal globally (Samuels and Szabadi 2008a, 2008b; Sara and Bouret 2012). Phasic LC‐NE activity has been associated with transient pupil dilation responses (PDRs), providing a noninvasive proxy for tracking adaptive neural responses to shifts in context (Murphy et al. 2011; Murphy et al. 2014; van Kempen et al. 2019). In particular, NE release is hypothesized to facilitate the updating of predictive models. When a violation or shift in the predictive model occurs, it generates “unexpected uncertainty” (Dayan and Yu 2006), indicating that events exceed expected uncertainty thresholds and necessitate adaptive model adjustments, to which the LC‐NE system is particularly sensitive. These theoretical accounts distinguish between uncertainty that is reducible (e.g., volatility‐driven) and uncertainty that is irreducible (e.g., stochastic randomness) (Piray and Daw 2021; Yu and Dayan 2005). In sensory contexts, irreducible uncertainty can arise from outliers within an otherwise stable environment, whereas reducible uncertainty reflects sustained changes in statistical structure—such as transitions between contextual states that signal informative change. The LC‐NE system is thought to be especially responsive to volatility‐driven uncertainty, as it reflects informative change that warrants updating internal models (Bianco et al. 2020). This phasic LC‐NE activation, in turn, triggers pupil dilation and facilitates the integration of bottom‐up signals by temporarily interrupting top‐down processes, thereby enabling the brain to detect emerging regularities in the environment and adapt efficiently (Bianco et al. 2019; Dayan and Yu 2006; O'Reilly 2013; Sara and Bouret 2012; Silvestrin et al. 2021; Zhao et al. 2019).

Crucially, the brain must not only respond to local prediction errors but also interpret them as potential evidence of broader changes in the environment's structure. When the regularity of a sequence suddenly shifts—transitioning from one coherent pattern to another—the accumulating mismatches may indicate that the existing model is no longer valid. Reflecting this process, abrupt violations of an established regularity trigger a robust pupillary response, signaling a possible recalibration of the predictive model (Bianco et al. 2020; Milne et al. 2021; Zhao et al. 2019). By contrast, when a new pattern emerges gradually, pupil dilation remains relatively muted, suggesting that incremental error accumulation can revise the existing framework without necessitating an abrupt shift (Bianco et al. 2020; Milne et al. 2021; Zhao et al. 2019). However, the precise mechanisms governing these different responses—and whether they indeed reflect a fundamental abandonment versus a revision of the previous predictive model—remain unclear. To address this gap in the literature, local deviations (at the stimulus level) and broader contextual shifts (signaling the need for a global model update) need to be examined together while distinguishing their associated papillary (dilation) responses. Notably, pupillometry offers a simple means to track these conditions, providing insight into how transitions in internal models unfold.

**FIGURE 1:**
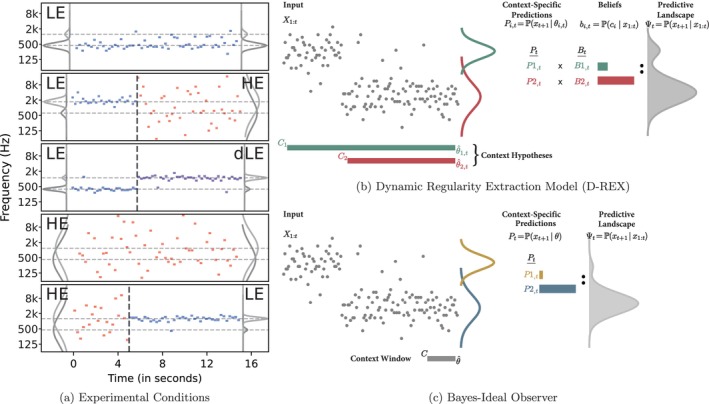
Quantifying short‐term expectations and contextual shifts in auditory sequences. (a) Tonal sequences (presented at 4 Hz, 200 ms on) were structures to shift between states of low entropy (LE) and high entropy (HE). Tones were drawn from two Gaussian distributions, with the sampling probability of each distribution and the sharpness of the distributions varying by condition. In the LE condition, tones were sampled from a narrow distribution (*σ* = 0.2 ± 0.15 octave jitter), with a high probability (90% ± 5% jitter) of being sampled from a single source. In the HE condition, tones were sampled from a broad distribution (*σ* = 1.85 ± 0.15 octave jitter), with equal probability (50% ± 5% jitter) of being drawn from either source. (b) Computational modeling was used to quantify tone‐by‐tone surprisal and expectations within tonal sequences. The D‐REX model (Skerritt‐Davis and Elhilali 2021) processes tonal sequences to generate a dynamic predictive landscape (Ψ_*t*_). It uses multiple context hypotheses, characterized by varying context lengths, to account for unknown changes in the observed sequence. Context‐specific predictions (*P*_*i*,*t*_ = P(*x*_*t*+1_ | *θ*_*i*,*t*_)) are informed by statistics (*θ*_*i*,*t*_) and weighted by their corresponding beliefs (*b*_*i*,*t*_ = P(*c*_*i*_ | *x*_1:__*t*_)). By combining these weighted predictions, the model yields the predictive distribution Ψ_*t*_ = P(*x*_*t*+1_ | *x*_1:*t*_), representing the probabilistic expectation of the next tone. (c) The Bayesian ideal observer operates similarly to the D‐REX model but with a fixed context window (set to 20 stimuli). This model computes context‐specific predictions (*P*_*t*_ = P(*x*_*t*+1_ | *θ*)) using a single, static context framework, forming the predictive landscape (Ψ_*t*_ = P(*x*_*t*+1_ | *x*_1:__*t*_)) based on a multi‐Gaussian distribution without dynamically adjusting to shifts in sequence structure.

In this study, we examined the relationship between pupil‐linked arousal and the interplay between two predictive metrics: surprisal—which quantifies how unexpected an event is, and precision—which reflects the stability or reliability of predictions within a context, akin to entropy. Specifically, we investigated how tone‐by‐tone surprisal and precision influence pupil dilation in response to stochastic auditory tone sequences. These tonal sequences were constantly shifting between states of high and low entropy (see Figure 1a). Using computational models of Bayesian inference, we quantified tone‐by‐tone surprisal and precision to capture the informational structure of the environment and its potential effects on a listener's arousal. We used a time‐resolved linear regression approach to estimate temporal response functions (TRFs), capturing how surprisal and precision modulate pupil dilation over time. We employed both a Dynamic Regularity Extraction (D‐REX) model, which adaptively integrates multiple context hypotheses to generate a predictive distribution (see Figure 1b), and a Bayesian ideal observer model, which assumes an optimal fixed‐length context window—ensuring that our results do not depend on a specific assumption about contextual integration (see Figure 1c). Beyond individual tone responses, we also explored pupil responses at context boundaries—where the entropy of the auditory environment shifted, such as during the transition from a low‐entropy (LE) to a high‐entropy (HE) environment, and vice versa. By removing the contributions of tone‐by‐tone surprisal to pupil response, we aimed to highlight the effect of broader arousal responses triggered by shifts in contextual stability, independently from transient prediction errors associated with individual tones. Specifically, transitions from low‐ to high‐entropy contexts (LE‐HE) reflect the loss of a predictive model and therefore a shift to irreducible uncertainty. Transitions from high‐ to low‐entropy contexts (HE‐LE) capture gradual, reducible change as stable structure begins to (re‐)emerge. Finally, transitions between distinct low‐entropy contexts (LE‐dLE) reflect volatility‐driven violations, where one established model is rapidly replaced by another, engaging reducible uncertainty during the transition. Through this approach, we aimed to provide insights into how the human brain implicitly integrates predictive metrics to aid rapid interpretation of auditory dynamic environments.

## 2 Results

We investigated pupil‐linked phasic arousal evoked by the interplay of tonal surprisal and precision in dynamically shifting auditory environments. We collected pupil dilation data from 28 participants, who passively listened to stochastic sequences of pure tones. Presented tones were sampled from two Gaussian distributions, with the sampling distribution and the sampling width varying in time. We aimed to quantify how much information was—in principle—available to listeners and to assess whether this information, captured in terms of surprisal, precision, and the information content of an auditory environment, could account for observed fluctuations in pupil‐linked phasic arousal response.

### 2.1 Phasic Pupil Responses Resolve Tonal Surprisal and Precision Variation in Dynamic Auditory Environments

To estimate surprisal and precision on a tone‐by‐tone basis, we modeled listeners as Bayesian ideal observers who form probabilistic predictions of the next tone given the tone history. Analyses were based on data from 24 of the 28 recruited participants (see Section 4.5 for details). Specifically, we employed the D‐REX model, a Bayesian inference framework that captures how listeners may update their beliefs about upcoming events (Skerritt‐Davis and Elhilali 2021). Surprisal was operationalized as the negative log probability of the presented tone, capturing how unexpected a given tone was, given the reliability (precision) of the predictive distribution, while precision was defined as the inverse of the variance over the prior probability distribution, indicating the listeners' confidence in its predictions. We used both metrics—surprisal and precision—as normative measures of how much information was available to listeners. We used a time‐resolved linear regression approach (see Section 4.7 for details) to assess the temporal dynamics of pupil dilation by estimating a TRF of the model‐induced pupil change. Each TRF combines the time‐lagged coefficients for one (model) regressor, providing a continuous estimate of how graded changes in surprisal or precision shape pupil dilation over time. Note that surprisal and precision regressors were fitted simultaneously, allowing us to partition the effect that each has on the pupil response. Although surprisal is defined within the precision‐weighted probability landscape, their empirical correlation was modest (*r* ≈ −0.24), indicating that they capture largely distinct aspects of the model's predictions. The resulting TRFs represent an impulse response showing how parametric variations in surprisal and precision translate into pupil dilation over time, revealing both the timing and strength of their influence. Within this framework, we asked (1) whether tonal surprisal and model precision drive phasic pupil responses, and (2) what the temporal dynamics of these responses are.

**FIGURE 2:**
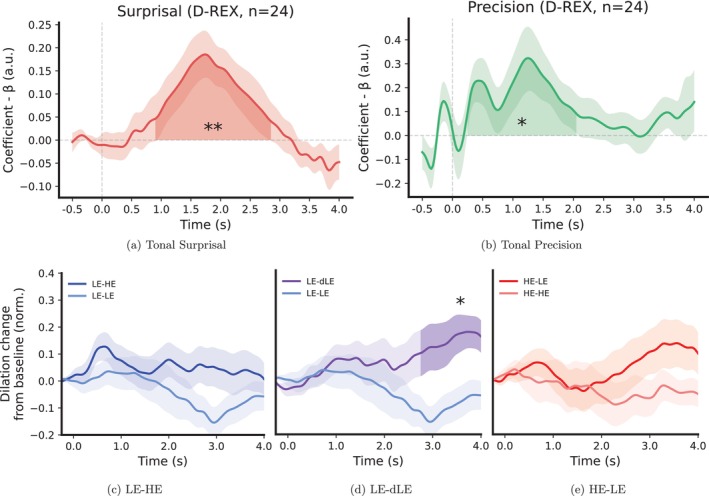
Expectation‐related pupil responses. The top row displays temporal response functions (TRFs) derived from time‐resolved regressions, while the bottom row shows event‐related averages aligned to condition boundaries. Top row: (a) shows the influence of tone‐by‐tone surprisal on pupil dilation, and (b) shows the influence of tone‐by‐tone precision. Robust pupil dilation responses to both tonal surprisal and precision across time indicate that the unexpectedness and confidence in predictions significantly modulate phasic arousal. In both, lightly shaded areas indicate bootstrapped 95% confidence intervals, while darkly shaded areas mark time windows of significant effects tested using cluster‐based permutation testing (Bonferroni‐adjusted *p* < 0.05). Bottom row: (c) depicts the transition from a low entropy (LE) to high entropy (HE), (d) the transition between distinct low‐entropy environments (LE to dLE), and (e) the transition from high entropy (HE) to low entropy (LE). Only transitions between distinct low‐entropy contexts (LE‐dLE) elicited a modest pupil dilation at the boundary, whereas transitions from low‐ to high‐entropy (LE‐HE) or high‐ to low‐entropy (HE‐LE) did not. Here, lightly shaded areas indicate bootstrapped standard errors across participants, while darkly shaded regions highlight time windows of significant differences from their respective control, tested using cluster‐based permutation testing (Bonferroni‐adjusted *p* < 0.05). For visualization, the pupil time courses were cropped to a 4‐s window following the boundary, matching the analysis window used for the TRFs.

The TRF of the surprisal regressor, derived from the D‐REX model, exhibited a strong sustained dilation between 900 ms and 2.8 s (*p* < 1*e−*4; Bonferroni‐corrected; see Figure 2a). This surprisal‐driven dilation response is consistent with previous reports, as summarized in Zekveld et al. 2018. The TRF of the precision regressor showed an immediate rise in pupil dilation with a first positive peak around 450 ms and a second positive dilation peak around 1.4 s (250 ms to 2 s, *p* < 1*e−*4; Bonferroni‐corrected; see Figure 2b). These results align with Silvestrin et al. (2021), which showed that pupil dilation reflects automatic learning of auditory stimulus precision.

To facilitate interpretability of how pupil responses differ between low‐ and high‐surprisal events, we divided model‐based tone surprisal into four bins, each containing an equal number of trials, and compared—using event‐related averaging—the pupil response in the upper and lower quartiles. This analysis confirmed the TRF findings for surprisal: High‐surprisal events elicit more papillary dilation compared to low‐surprisal events (see Supplement—Figure S1). For surprisal, event‐related averaging is meaningful because its low autocorrelation (*r* ≈ 0.48) reflects a tone‐by‐tone dynamic, where effects of successive responses cancel out. Precision, by contrast, evolves gradually (*r* ≈ 0.98) with shifts that persist across many tones while also shaping the likelihood of surprising events. Event‐related averaging would blur these effects, making the TRF approach better suited to capture their dynamics.

Finally, we replicated our regression results using a simpler ideal observer model with a fixed context window as a generative model predicting a mixture of two Gaussians (see Section 4.6.2 for details). This replication allowed us to verify whether the observed pupil dynamics were consistent across models differing in their assumed temporal scope of contextual integration (Skerritt‐Davis and Elhilali 2021). The resulting TRFs for surprisal and precision, derived from this ideal observer model, exhibited dynamics similar to those obtained with the D‐REX model, indicating that our findings are robust to the specific form of inference (see Supplement—Figure S2).

### 2.2 Phasic Pupil Responses Track Context Shifts, Not Regularity Emergence or Violations

We then explored how shifts in perceived auditory context influence arousal‐driven pupil responses, particularly at the boundaries of model transitions. Analyses were based on data from 22 of the 28 recruited participants (see Section 4.5 for details). These boundaries include transitions from a low‐entropy to a high‐entropy environment, from high‐ to low‐entropy environment, or shifts between two distinct low‐entropy environments. A low‐entropy environment was characterized by tones sampled from a narrow distribution with a higher probability of sampling from one of the two sources, while a high‐entropy environment involved tones sampled from broad distributions with approximately equal probabilities of sampling from either source. To ensure that the observed pupil responses reflected the emergence, violation, or shifts in perceived context at the boundaries—rather than being confounded by tonal surprisal effects—we first regressed out the variance explained by tonal surprisal based on the previously presented regression analysis. Because low‐ and high‐entropy contexts differ in their surprisal profiles, this provided a more stringent control, leaving a residual signal reflecting context model shifts rather than the individual surprising tones that constitute those contexts. We also include the analysis on the full preprocessed pupil responses without regressing out tonal surprisal (see Supplement—Figure S3) to demonstrate that the results do not hinge on this additional step.

We observed significant PDRs during transitions between auditory contexts characterized by changes in model structure. Specifically, transitions between distinct low‐entropy environments (LE‐dLE) elicited significant PDRs, indicative of heightened arousal after these context boundaries (2.5–4.6 s, *p* = 0.04 Bonferroni‐corrected, *d* = 0.63; see Figures 2d and S4b). Surprisingly, transitions from a low‐entropy to a high‐entropy environment (LE‐HE)—in other words, the violation of a model—did not result in a significant PDR (2.8–3.0 s, *p* = 1.0 Bonferroni‐corrected, *d* = 0.47; see Figures 2c and S4a). Additionally, transitions from high‐ to low‐entropy environments (HE‐LE) did not elicit significant PDRs (2.9–5.9 s, *p* = 0.21 Bonferroni‐corrected, *d* = 0.35; see Figures 2e and S4c). While these results indicate that transitions between distinct low‐entropy contexts (LE‐dLE) are sufficient to modulate arousal, the overall effect size for these transitions was relatively modest, given the inherent variability and limited temporal precision of pupillary measures. Furthermore, our primary analyses show that tone‐by‐tone surprisal and tonal precision (i.e., the strength of the auditory model) remained central factors in shaping pupil responses. Taken together, these results suggest that while the degree of context stability and model strength (as captured by tone‐by‐tone surprisal and precision) influence arousal dynamics, this influence is not necessarily translated to phasic pupillary responses at the boundary.

## 3 Discussion

We investigated whether and how pupil dilation is modulated by surprisal and precision during the processing of dynamic auditory environments, and how it changed in response to context boundaries. We combined computational models of Bayesian inference with time‐resolved regressions of pupil dilation data to examine how humans adaptively respond to shifts in unexpectedness and stability within these probabilistic auditory environments.

The current results suggest robust phasic PDRs to both surprisal and precision during tone‐by‐tone auditory sequence processing. Specifically, graded surprisal responses were associated with a sustained pupil dilation beginning around 900 ms after tone onset and continuing up to 2.8 s. This response highlights how even momentary deviations from statistical expectations evoke prolonged pupillary responses. Similarly, graded precision responses—quantified as the inverse of variance in probabilistic predictions—elicited a pupil response starting as early as 450 ms, peaking at 1.4 s, and subsiding by 2 s. The difference in timing suggests that precision engages arousal earlier but more briefly, whereas surprisal is associated with a later arousal response that extends over a longer interval. These findings indicate that both *surprisal* and the stability of environmental regularities (*precision*) are tightly coupled with arousal dynamics, consistent with the proposed role of the locus coeruleus–norepinephrine (LC‐NE) system in facilitating adaptive model updates (Bianco et al. 2019, 2020; Dayan and Yu 2006; Murphy et al. 2011; Murphy et al. 2014; O'Reilly 2013; Sara and Bouret 2012; van Kempen et al. 2019). Furthermore, transitions between distinct low‐entropy auditory environments (LE‐dLE) elicited significant PDRs, while transitions from low‐entropy to high‐entropy environments (LE‐HE) or from high‐ to low‐entropy environments (HE‐LE) failed to evoke such responses. This pattern suggests that when the environment shifts from one low‐entropy (i.e., narrow, more predictable) context to another distinct low‐entropy context, the resulting boundary represents a more salient model transition, triggering a stronger arousal response. In contrast, transitions between predictable (low‐entropy) and less predictable (high‐entropy) contexts may dilute the saliency of a boundary, as high‐entropy states are inherently more diffuse and harder to differentiate from other contexts. Thus, within these dynamic auditory environments, the clarity and stability of context transitions (as in LE‐dLE) drive boundary‐related arousal responses more strongly than the mere increase or decrease in statistical entropy (LE‐HE or HE‐LE).

During the experiment, participants likely acquired an implicit understanding of the statistical stimulus properties—such as sampling means, distribution widths, and probabilistic mixtures—which shaped their expectations and uncertainty. Although the Bayesian models used offer a principled way to quantify the statistical structure of the environment, they assume ideal inference rather than participants' exact perceptual strategies, which were not explicitly assessed in this study by means of a behavioral task. This limitation reflects the challenge of probing implicit statistical learning without imposing explicit task demands. In this context, pupillary changes are thought to reflect arousal responses to unexpected uncertainty (Dayan and Yu 2006). Since we observed consistent pupil responses related to tonal surprisal, we conclude that these changes in the environment are continuously implicitly monitored and integrated, even when they lie at the fringes—but still within—the current predictive model. This suggests an ongoing monitoring process of adaptive refinement, where the brain integrates (subtle) deviations to adjust its internal model, supporting the dynamic and flexible nature of predictive processing.

The faster, more transient pupil response to precision compared to surprisal suggests that listeners are not only reacting to unexpected events but also actively access the stability of their predictive models. However, the pupil responses linked to precision showed a rhythmic modulation around 250 ms, plausibly reflecting residual autocorrelation in the precision regressor associated with the regular 4‐Hz stimulus timing. Although our time‐shifted FIR model explicitly accounts for temporal autocorrelation across tones within the TRF window, rapid precision transients time‐locked to each tone onset (< 250 ms) at high autocorrelation cannot be fully separated and may therefore superimpose on and obscure genuine precision‐related activity. This rhythmic component reduces the interpretability of the finer waveform structure within the precision response, but does not undermine the broader temporal dissociation observed between precision (earlier, more transient) and surprisal (later, sustained) effects. In the precision analysis, this effect is more pronounced due to the higher temporal autocorrelation of the precision regressor relative to the surprisal regressor. Despite this, the dual sensitivity to surprisal and precision demonstrates an ongoing balance between reacting to transient changes and the maintenance of stable predictive models. These results are in line with previous research (Bianco et al. 2019, 2020; Silvestrin et al. 2021), which showed that deviant tones within narrower distributions elicited stronger pupil dilation, suggesting that listeners track distributional variance even when it is task‐irrelevant. Extending these observations, the current study reveals that pupil responses reflect a range of prediction errors—from subtle deviations to larger outliers—enabling the brain to distinguish between local fluctuations within a stable context and evidence of broader context changes.

In addition to tone‐by‐tone responses, we examined pupil responses to broader contextual shifts. Transitions between two distinct informative (low‐entropy) environments elicited significant PDRs, whereas transitions from low‐ to high‐entropy environments or from high‐ to low‐entropy environments did not. We propose that transitions between distinct informative contexts may provide stronger evidence for a model shift than transitions involving a shift between an informative and a non‐informative context, as such abrupt changes warrant immediate model updates. For instance, transitions between two distinct informative contexts may signal the need to update the accumulated internal model, prompting an arousal response. This response likely reflects heightened sensitivity to unexpected uncertainty, as the system adapts to an abrupt change in the underlying structure of the environment. In line with theoretical accounts distinguishing between reducible (volatility‐driven) and irreducible (stochastic) uncertainty (Piray and Daw 2021; Yu and Dayan 2005), the LE‐dLE condition entailed the largest volatility change, thus evoking a more pronounced arousal response and signaling the need to update the internal model. In contrast, given the probabilistic nature of the stimuli used here, transitions involving high‐entropy contexts can still be accommodated within the current model—though at its limits—as the subsequent tones remain at least partially consistent with the underlying probabilistic structure. This may explain the differences between the current findings and those of previous studies (Milne et al. 2021; Zhao et al. 2019), where unexpected uncertainty was introduced more abruptly, triggering stronger arousal responses. Unlike regular environments, where violations are sharply defined, the probabilistic nature of these auditory sequences allowed participants to anticipate variability within certain bounds, blurring the boundary between contexts and requiring more evidence accumulation to detect and respond to a shift. Thus, transitions to or from high‐entropy contexts demand more sampling over time to gather sufficient evidence for a model violation—as the per‐tone evidence is weak and the resulting pupil signal becomes temporally smeared—making the boundary between contexts less apparent. Future work could test this hypothesis by manipulating stimulus timing to examine how temporal structure affects evidence accumulation and arousal responses. These findings tentatively suggest that while the LC‐NE system is generally sensitive to violations of regularity models, it primarily responds to transitions where evidence for model change is strongest.

Our findings provide novel insights into the interplay between surprisal, precision, and contextual shifts in shaping arousal responses. Beyond demonstrating the LC‐NE system's role in monitoring for model change, we show that this arousal mechanism remains active even during passive listening, continuously monitoring for deviations and evaluating the strength of the current predictive model. This suggests that listeners are perpetually preparing for potential future changes, ensuring adaptability in uncertain and ever‐changing perceptual environments.

Future studies should further disentangle the relative contributions of surprisal and precision to context boundary effects by directly comparing pre‐ and post‐boundary responses or manipulating transition saliency. Additionally, deterministic environments could be used to investigate when the LC‐NE system becomes less active, such as when listeners rely more on memory or when priors are sharp enough to render most stimuli unsurprising. These studies could help clarify when and why the LC‐NE system prioritizes change monitoring and when it allows for greater model stability.

In conclusion, this study demonstrates the dynamic interplay between prediction, uncertainty, and arousal in auditory contexts. By leveraging Bayesian inference models and time‐resolved pupil analyses, we showed how the human brain continuously monitors and adapts to its sensory environment. These findings emphasize the role of an always‐active arousal mechanism in supporting predictive processing and model updating, offering new insight into how humans navigate complex and probabilistic sensory environments.

## 4 Methods

### 4.1 Participants

Twenty‐eight participants (23 female, M = 21.6 years old, range = 18 to 35 years old) participated in the experiment. All participants reported normal or corrected‐to‐normal vision and normal hearing, as well as no history of neurological disorders. The study was approved by the Ethics Review Committee for Psychology and Neuroscience (ERCPN; Approval Code: OZL‐232‐01‐01‐2021) at Maastricht University, following the principles expressed in the Declaration of Helsinki.

### 4.2 Experimental Setup

Participants were seated 50 cm from a monitor (iiyama ProLite B2483HSU; 1920 × 1080‐px resolution, 60‐Hz refresh rate) in a quiet, dimly lit room. A chin rest (Arrington HeadLock) was used to stabilize their head position. Pupil dilation was recorded monocularly from the right eye using an Arrington ViewPoint EyeTracker 400 (225‐Hz sampling rate), positioned below the monitor. Data collection was managed via Arrington ViewPoint software (Arrington 1997). The pupil area was used as a stable proxy for pupil dilation. Audio stimuli were delivered through USB headphones.

Before the experiment, a standard 16‐point calibration was conducted to ensure accurate eye tracking. During the trials, participants were instructed to maintain focus on a central fixation point displayed against a mean‐luminance gray background. Participants were instructed to minimize blinking and limit head movements throughout stimulus presentation to reduce artifacts.

To ensure consistent perceived loudness across the frequency range, participants completed a loudness equalization task. In this task, they compared pairs of tones sampled from the target range, and the responses were used to generate an equal‐loudness curve for each participant (Suzuki and Takeshima 2004). This curve was then applied to adjust the intensity of the tones used in the experiment.

### 4.3 Experimental Design

The experiment lasted approximately 1.5 h and consisted of nine blocks, each containing 16 trials, for a total of 144 trials per participant. Each trial was divided into two segments, one occurring before and one after a boundary. Participants engaged in a passive listening task, with no visual or auditory feedback provided. Pure tones were presented at a rate of 4 Hz, each lasting 200 ms with a 50‐ms intertone silent period.

#### 4.3.1 Stimuli

Stimuli alternated between periods of stability and high entropy within the predictive auditory landscape. Tones were sampled with replacement from one of two Gaussian distributions, with center frequencies ranging from 500 Hz to 3 kHz. To ensure the distributions were sufficiently distinct, the center frequencies were separated by a minimum of 1.3 octaves. Sampled tone frequencies were also constrained to discrete octave steps by rounding to the nearest 1/12th of an octave (*log*2‐space). The width of the distributions and the probability of sampling from either of the two distributions varied depending on the experimental condition.

Each trial consisted of pre‐ and post‐boundary conditions, separated by a boundary. To reduce temporal predictability, the boundary occurred after 20 tones with a jitter of ±5 tones (±1.25 s). The post‐boundary condition consisted of 40 tones, resulting in a total trial length of 60 tones (15 s). Boundaries reflected only a change in the underlying tone‐sampling distribution, with all other acoustic parameters (tone rate, duration, and intensity) held constant. Each trial was followed by an intertrial interval of 6 s. After every nine trials, participants were given the opportunity to take a short break before continuing. Pre‐boundary conditions were either low entropy (LE) or high entropy (HE). Low‐entropy environments consisted of tones sampled from a narrow distribution (*σ* = 0.2 ± 0.15 oct jitter), with a high probability of sampling from one source (90% ± 5% jitter). In contrast, high‐entropy environments involved tones sampled from a broad distribution (*σ* = 1.85 ± 0.15 oct jitter), with equal probabilities of sampling from either source (50% ± 5% jitter). Post‐boundary conditions included the same environments as pre‐boundary conditions, with the addition of a differently informative environment (differently low‐entropy, dLE), where the most probable distribution of the low‐entropy environment was swapped. Boundaries involved transitions from low‐ to high‐entropy (LE‐HE), high‐ to low‐entropy (HE‐LE), or low‐entropy to different distinct low‐entropy (LE‐dLE). Trials without transitions included no‐transition low entropy (LE‐LE) and no‐transition high entropy (HE‐HE). In no‐transition trials (LE–LE and HE–HE), tones were drawn from a single unchanged distribution throughout the trial.

The resulting five conditions (LE‐LE, LE‐HE, LE‐dLE, HE‐LE, and HE‐HE), each consisting of 24 trials, were presented in random order. The experiment was designed using Psychophysics Toolbox extensions (Brainard 1997; Kleiner et al. 2007; Pelli 1997) in MATLAB‐R2019b (The MathWorks 2019).

### 4.4 Preprocessing

All preprocessing steps were conducted using the FieldTrip toolbox (Oostenveld et al. 2011) in MATLAB (The MathWorks 2019). To address the nonfixed sampling rate in the eye tracker recordings, a time‐accurate downsampling from 225 to 125 Hz was performed as an initial preprocessing step. The pupil data were first smoothed using a Hanning window with a length of 150 ms. Eye blink artifacts were detected by identifying rapid changes in the signal amplitude (using ft_artifact_zvalue; Oostenveld et al. 2011). Following each detected blink, an additional 200 ms after the blink offset was removed to minimize artifacts caused by the recapturing of the pupil by the eye tracker.

To address the gaps in the data caused by blink removal, a piecewise cubic Hermite interpolation was performed to reconstruct the missing values in a time‐consistent manner (using ft_interpolatenan; Oostenveld et al. 2011). The data were then downsampled to 40 Hz for further analysis. A bandpass filter (0.1 to 4 Hz) was subsequently applied to de‐trend the data and remove high‐frequency fluctuations (using ft_preprocessing; Oostenveld et al. 2011).

#### 4.4.1 Blink Regression

To further account for post‐blink effects on pupil size (Mathôt et al. 2018), we used a regression‐based approach. Specifically, a sparse array was created with impulses aligned to blink offsets, which were thereafter convolved with the average pupil dilation observed in the 1.6 s following each blink. This convolved array served as a regressor in a linear model, with the residuals providing a blink‐artifact‐reduced proxy for pupil dilation.

### 4.5 Data Exclusion Criteria

Trials were excluded if 35% or more of the data within a trial were interpolated, or if there was a gap of 1.5 s or longer with no data. On average, this resulted in the exclusion of 8% of trials (11.7 out of 144 trials on average, SD = 15.78 trials). For the time‐shifted regression analysis, data from four participants were excluded due to more than 50% of their trials being removed. This analysis treats each trial as a continuous sequence, so the selection criterion only concerns the overall number of available trials, irrespective of condition. Data from 24 participants were included in this analysis. In the condition‐based analysis, by contrast, trial counts within each condition matter. Participants were excluded if any condition contained fewer than 13 remaining trials (i.e., more than 50% missing). Consequently, data from 22 participants were included in this analysis.

### 4.6 Computational Modeling

Contextual tone‐based predictors were formed using inference models—models computing the probability of each tone given the preceding tones. Here, we used the D‐REX model (Skerritt‐Davis and Elhilali 2021), which is a Bayesian inference computation model for predictive processing in auditory perception of sequential sounds. We also built our own Bayesian Ideal Observer model, which estimated the predictive space relying only on Bayesian optimal inference to create a Gaussian mixture predictive landscape.

#### 4.6.1 D‐REX

The D‐REX model employs Bayesian sequential prediction with perceptual constraints, such as memory (m) and observation noise (n), to simulate the brain's processing of sound sequences over time. Memory (m) restricts the number of prior time points considered; we set *m* to remain within the boundaries of a trial, while observation noise (n) introduces Gaussian noise to the input. The model continuously predicts the distribution of the next time point ($x_{t}+1$) based on previous observations ($X_{1}$) by estimating local statistics ($\hat{\theta }$), which correspond to the sample mean and sample variance of the input.

In addition to these features, the model can incorporate a Gaussian mixture model (GMM) to represent multiple components present within the sound sequences. For our stimuli specifically, we set the maximum to two components, corresponding to our two center frequencies. To manage the creation of new components within the GMM, a threshold parameter, beta, was set to 0.2. This beta value, multiplied by a scaling factor for predictive probabilities, also helps to limit the overall stimuli space.

The model accounts for possible changes in parameters ($\hat{\theta }$) by generating hypotheses across varying run lengths (rt) and integrating these to predict future observations:

$$P\left(x_{t+1}|x_{1:t}\right)=\sum_{r_{t}}P\left(x_{t+1}|r_{t},x_{t-r_{t}+1:t}\right)P\left(r_{t}|x_{1:t}\right)$$

The primary output of the model is surprisal ($S_{t}+1$), representing the discrepancy between the predicted probability and the actual observation:

$$S_{t}+1=-\log P\left(x_{t}+1|x_{1}:t\right)$$

A lower probability event results in higher surprisal, with zero surprisal indicating a perfectly predicted event.

In addition to surprisal, the model provides a measure of precision, derived from the probability landscape of each stimulus. Precision, defined as the inverse of the variance within this inferred landscape, quantifies the stability of the model's predictions. Higher precision indicates greater confidence and stability in the predictions, while lower precision reflects increased uncertainty and variability.

$$\text{Precision}\left(t+1\right)=\frac{1}{\sum_{r_{t}}P\left(r_{t}|x_{1:t}\right)\;\left(\sigma_{r_{t}}^{2}+mu_{r_{t}}^{2}\right)-{\left(\sum_{r_{t}}P\left(r_{t}|x_{1:t}\right)\;\mu_{r_{t}}\right)}^{2}}$$

Here, $P\left(r_{t}|x_{1:t}\right)$ is the posterior probability of a given run length $r_{t}$ given the observations up to time t; $\mu_{r_{t}}$ and $\sigma_{r_{t}}$ are the predicted mean and standard deviation, respectively, of the next stimulus under run length $r_{t}$.

#### 4.6.2 Bayesian Ideal Observer

The ideal observer model is a theoretical framework that predicts how a Bayes‐optimal observer would perceive and interpret sensory information. In this case, the model is applied to a sequence of auditory stimuli, aiming to estimate the underlying probability landscape representing the perceived distribution of presented tones over time. The model assumes that tone frequency data can be described by a mixture of Gaussian distributions, each representing different perceptual categories of stimuli. The model estimates the parameters of these Gaussian components—means (μ), variance ($\sigma^{2}$), and weights (w)—as the observer accumulates evidence from the presented stimuli. For each stimulus, the model predicts its probability within the GMM landscape. Given a mixture of *n* Gaussian components, the probability of observing a stimulus $X_{t}$ at time t is computed as follows:

$$P\left(x_{t}\right)=\sum_{i=1}^{n}w_{i}\left[\Phi \left(\frac{x_{t}+\frac{\mathrm{res}}{2}-\mu_{i}}{\sigma_{i}}\right)-\Phi \left(\frac{x_{t}+\frac{\mathrm{res}}{2}-\mu_{i}}{\sigma_{i}}\right)\right]$$

Here, $w_{i}$ is the weight of the *i*‐th Gaussian component, $u_{i}$ is its mean, $o_{i}$ is its standard deviation, and $\Phi \left(\cdot \right)$ is the cumulative distribution function (CDF) of the standard normal distribution. The resolution (res) parameters are set to 1/12, reflecting the delta resolution of the stimulus space. This probability indicates the likelihood of the observer encountering the stimulus $x_{t}$ given the current estimate of the underlying frequency distribution. The model uses an optimal context window of 20 stimuli, meaning that each probability estimate is informed by up to 20 preceding stimuli.

To determine the optimal context window for the ideal observer model, we evaluated the model's performance across a range of context window sizes (4 to 50 stimuli). Performance was quantified using the log‐likelihood of the observed data, calculated as the sum of the logarithms of the predicted probabilities for each stimulus. The context window that maximized the log‐likelihood was selected as the optimal size, representing the window that best captured the underlying probabilistic structure of the stimuli.

The model also calculates the *surprisal* of each stimulus, quantifying how unexpected the stimulus is given its predicted probability. The surprisal $S\left(x_{t}\right)$ is defined as follows:

$$S\left(x_{t}\right)=-\log P\left(X_{t}\right)$$

This surprisal value measures the unpredictability of each stimulus, with higher values indicating more surprising stimuli.

The model also calculates *precision*, which reflects the reliability of predictions within the predictive landscape. Precision is defined as the inverse of the variance within the predictive landscape. Higher precision indicates a more stable and reliable predictive landscape, while lower precision suggests greater uncertainty.

$$\text{Precision}=\frac{1}{\sum_{i=1}^{n}w_{i}\;\left(\sigma_{i}^{2}+\mu_{i}^{2}\right)-{\left(\sum_{i=1}^{n}w_{i}\;\mu_{i}\right)}^{2}}$$

Here, $w_{i}$ is the weight of the *i*‐th Gaussian component, satisfying $\sum_{i=1}^{n}w_{i}=1$; $\mu_{i}$ and $\sigma_{i}$ are the mean and standard deviation of the *i*‐th Gaussian component, respectively; and X represents a random variable drawn from the mixture distribution.

### 4.7 Finite Impulse Response (FIR) Model

We applied time‐resolved linear regression to the pupil data to explore the temporal dynamics of tonal surprise and precision, employing a time‐shifted FIR model (rERP; N. J. Smith and Kutas 2015). This method enabled us to disentangle the responses to different tonal features and correct for their temporal overlap. Each feature of interest was modeled as an impulse regressor with one continuous value per tone (xϵR). The pupil data was z‐scored separately for each *trial*. Regressors were normalized to range from 0 (minimum possible value) to 1 (maximum possible value).

We constructed a time‐expanded regression matrix *M*, which included time‐shifted versions of each regressor arranged column‐wise (*t*_*min*_ = −0.5 s, *t*_*max*_ = 4.0 s relative to tone onset, resulting in 366 columns per regressor based on the sampling rate of 40 Hz).

Next, we estimated the regression weights $\hat{\beta }$ using linear regression:

$$\hat{\beta }={\left(M^{T}M\right)}^{-1}M^{T}y$$

Collectively, these weights can be interpreted as a TRF, which depicts how features modulate over time (Crosse et al. 2016; Ding and Simon 2012). In practice, this means the TRF reflects how graded variations in a predictor are expressed in pupil dilation at different latencies.

#### 4.7.1 Cross‐Validation Procedure

All regression analyses were performed using cross‐validation on left‐out data. Valid trials that were not excluded were used to determine appropriate splits for cross‐validation, aiming for eightfold cross‐validation with a tolerance of twofold (i.e., between sixfold and tenfold). If the available data did not allow for an exact split, additional data were discarded to meet the target configuration. Cross‐validation was used to assess generalizability of the estimated TRFs, providing more reliable estimates of the effects of surprisal and precision.

### 4.8 Analysis

Pupil data were z‐scored for each trial individually to facilitate comparisons across trials, conditions, and participants. The z‐scored data were analysed using a time‐shifted regression approach to obtain TRFs for both surprisal and precision within a temporal window of 0.5 to 4 s. The regression coefficients obtained from this analysis were averaged across participants to derive a group‐level TRF function. To estimate variance, a bootstrapped 95% confidence interval (uncorrected) was calculated with 10,000 bootstrap samples. We then used cluster‐based permutation tests (10,000 permutations) to identify significant temporal clusters of effects relative to the baseline time window (−0.5 to 0 s). To determine significant time points, we used threshold‐free cluster enhancement (TFCE; Smith and Nichols 2009), and family‐wise error (FWE) correction was applied using the Bonferroni method, correcting for two TRFs.

In a separate analysis, pupil changes were examined at the segment change point within each trial across the five experimental conditions: HE‐LE, LE‐HE, LE‐dLE, HE‐HE, and LE‐LE. For each condition and participant, an event‐related average of pupil dilation was computed, with tonal effects for surprisal removed by first regressing out the variance explained by this factor in the time‐shifted analysis. This approach ensured that the residuals used in this second analysis were not confounded by tone‐driven effects after or at the boundary. A baseline correction, using the 10 tones (250 ms) prior to the boundary as the reference, was then applied. To identify significant temporal clusters after a context shift, TFCE was performed on conditions with a boundary (i.e., HE‐LE, LE‐HE, and LE‐dLE) compared to their respective controls (HE‐HE or LE‐LE). Specifically, three paired comparisons were conducted: HE‐HE vs. HE‐LE, LE‐LE vs. LE‐HE, and LE‐LE vs. LE‐dLE. These pairwise comparisons were performed within a window of 0–6 s after the boundary. To control for multiple comparisons across these three tests, Bonferroni correction was applied to adjust the resulting *p*‐values.

## Author Contributions

**Jorie J. G. van Haren:** conceptualization, data curation, formal analysis, investigation, methodology, software, validation, visualization, writing – original draft, writing – review and editing. **Jan‐Luca Schröder:** data curation, formal analysis, investigation, software, validation, writing – original draft, writing – review and editing. **Floris P. de Lange:** supervision, writing – review and editing. **Sonja A. Kotz:** supervision, writing – review and editing. **Federico De Martino:** funding acquisition, supervision, writing – review and editing.

## Funding

This work was supported by the European Research Council (ERC) under the European Union's Horizon 2020 research and innovation programme (Grant Agreement No. 101001270).

## Conflicts of Interest

The authors declare no conflicts of interest.

## Supporting information


**Figure S1:** Pupil responses to high‐ and low‐surprisal events. Surprisal values were divided into four equal‐width bins, and pupil responses were analyzed using event‐related averaging. Trial counts were equalized across bins. Shaded areas represent bootstrapped confidence intervals. (a) depicts pupil dilation responses for the lowest (blue) and highest (red) surprisal bins. (b) depicts the difference over time between high‐ and low‐surprisal bins.
**Figure S2:** Temporal response functions (TRFs) obtained from time‐resolved regressions, with the y‐axis representing coefficient values. Each subplot illustrates how tonal surprisal or tonal precision modulates pupil dilation over time. Lightly shaded areas indicate the bootstrapped 95% confidence interval, whereas darkly shaded areas denote significant deviations from baseline. (a) Observer Ideal Surprisal: Significant modulation between 300 ms and 2.8 s (*p* < 1e‐4, Bonferroni‐corrected). (b) Observer Ideal Precision: Significant dilation from 900 ms to 2.2 s (*p* = 1.2e‐3, Bonferroni‐corrected).
**Figure S3:** Boundary‐related pupil responses before accounting for tonal surprisal. Event‐related averages of pupil responses, comparing each condition to its corresponding control, time‐locked to the boundary (zero point). Lightly shaded areas indicate bootstrapped standard errors across participants, while darkly shaded regions highlight time windows where the condition significantly differed from control. (a) Transition from a low‐entropy (LE) to high‐entropy (HE) environment, no significant cluster found (2.9–3.0 s, *p* > 1.0, Bonferroni‐corrected). (b) Transition from a low‐entropy (LE) environment to a different—distinctly—informative environment (dLE), significant cluster found (2.5–3.0 s, *p* = 0.033, Bonferroni‐corrected). (c) Transition from a high‐entropy (HE) to a low‐entropy (LE) environment, no significant cluster found (2.9–5.9 s, *p* = 0.165, Bonferroni‐corrected).
**Figure S4:** Extended boundary‐related pupil responses across the full post‐boundary window. Extending the analysis to the complete post‐boundary segment (40 tones, corresponding to 10 s) did not meaningfully alter the results or interpretation. Event‐related pupil time courses are shown for all boundary transitions without temporal cropping. Lightly shaded regions denote bootstrapped standard errors across participants, while dark shading indicates significant time windows identified using cluster‐based permutation testing performed over the full 10‐s window (Bonferroni‐adjusted *p* < 0.05).

## Data Availability

All data, analysis scripts, and modeling code are available on the Open Science Framework (OSF)—https://osf.io/brjyq/?view_only=f6d6c483b42549d3bca617ae7b301531. This includes raw and preprocessed pupil data, stimulus files, model outputs, and code to reproduce all analyses.
