# Supplementary material for: Tonal Surprisal and Contextual Shifts Evoke Distinct Pupil Dilation During Dynamic Sound Sequences
Source: Eur J Neurosci. 2026 Jan 4;63(1):e70380. doi: 10.1111/ejn.70380 (PMC12765649; doi:10.1111/ejn.70380)
Supplement: Supplementary file 1 — Figure S1: Pupil responses to high‐ and low‐surprisal events. Surprisal values were divided into four equal‐width bins, and pupil responses were analyzed using event‐related averaging. Trial counts were equalized across bins. Shaded areas represent bootstrapped confidence intervals. (a) depicts pupil dilation responses for the lowest (blue) and highest (red) surprisal bins. (b) depicts the difference over time between high‐ and low‐surprisal bins. Figure S2: Temporal response functions (TRFs) obtained from time‐resolved regressions, with the y‐axis representing coefficient values. Each subplot illustrates how tonal surprisal or tonal precision modulates pupil dilation over time. Lightly shaded areas indicate the bootstrapped 95% confidence interval, whereas darkly shaded areas denote significant deviations from baseline. (a) Observer Ideal Surprisal: Significant modulation between 300 ms and 2.8 s (p < 1e‐4, Bonferroni‐corrected). (b) Observer Ideal Precision: Significant dilation from 900 ms to 2.2 s (p = 1.2e‐3, Bonferroni‐corrected). Figure S3: Boundary‐related pupil responses before accounting for tonal surprisal. Event‐related averages of pupil responses, comparing each condition to its corresponding control, time‐locked to the boundary (zero point). Lightly shaded areas indicate bootstrapped standard errors across participants, while darkly shaded regions highlight time windows where the condition significantly differed from control. (a) Transition from a low‐entropy (LE) to high‐entropy (HE) environment, no significant cluster found (2.9–3.0 s, p > 1.0, Bonferroni‐corrected). (b) Transition from a low‐entropy (LE) environment to a different—distinctly—informative environment (dLE), significant cluster found (2.5–3.0 s, p = 0.033, Bonferroni‐corrected). (c) Transition from a high‐entropy (HE) to a low‐entropy (LE) environment, no significant cluster found (2.9–5.9 s, p = 0.165, Bonferroni‐corrected). Figure S4: Extended boundary‐related pupil responses across [file EJN-63-0-s001.pdf]

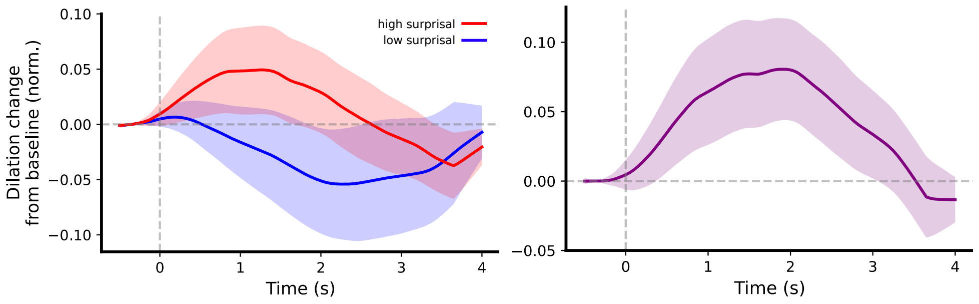

(a) Low vs. High Surprisal (b) High–Low Surprisal Difference

Figure S1: **Pupil responses to high- and low-surprisal events.** Surprisal values were divided into four equal-width bins, and pupil responses were analyzed using event-related averaging. Trial counts were equalized across bins. Shaded areas represent bootstrapped confidence intervals. **S1**a depicts pupil dilation responses for the lowest (*Blue*) and highest (*Red*) surprisal bins. **S1**b depicts the difference over time between high- and low-surprisal bins.

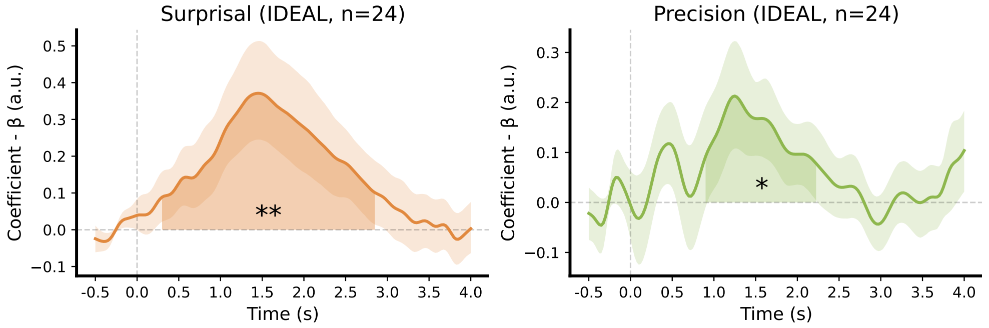

(a) Tonal Surprisal (b) Tonal Precision

Figure S2: **Temporal Response Functions (TRFs) obtained from time-resolved regressions**, with the y-axis representing coefficient values. Each subplot illustrates how tonal surprisal or tonal precision modulates pupil dilation over time. Lightly shaded areas indicate the bootstrapped 95% confidence interval, whereas darkly shaded areas denote significant deviations from baseline. **S2**a Observer Ideal Surprisal: Significant modulation between 300 ms and 2.8 s (p < 1e-4, Bonferroni corrected). **S2**b Observer Ideal Precision: Significant dilation from 900 ms to 2.2 s ($p = 1.2$e-3, Bonferroni corrected).

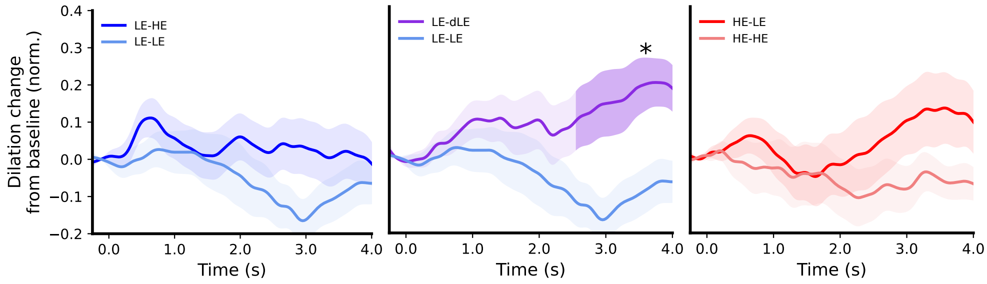

(a) LE-HE (b) LE-dLE (c) HE-LE

Figure S3: **Boundary-related pupil responses before accounting for tonal surprisal.** Event-related averages of pupil responses, comparing each condition to its corresponding control, time-locked to the boundary (zero point). Lightly shaded areas indicate bootstrapped standard errors across participants, while darkly shaded regions highlight time windows where the condition significantly differed from control. **S3**a Transition from a low-entropy (LE) to high-entropy (HE) environment, no significant cluster found (2.9–3.0 s, $p > 1.0$, Bonferroni corrected). **S3**b Transition from a low-entropy (LE) environment to a different - distinctly - informative environment (dLE), significant cluster found (2.5–3.0 s, $p = 0.033$, Bonferroni corrected). **S3**c Transition from a high-entropy (HE) to a low-entropy (LE) environment, no significant cluster found (2.9–5.9 s, $p = 0.165$, Bonferroni corrected).

(a) LE–HE

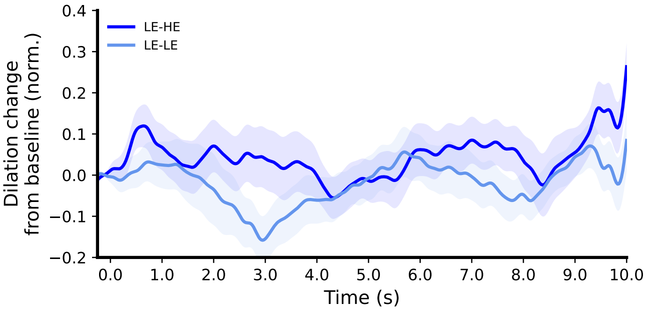

(b) LE–dLE

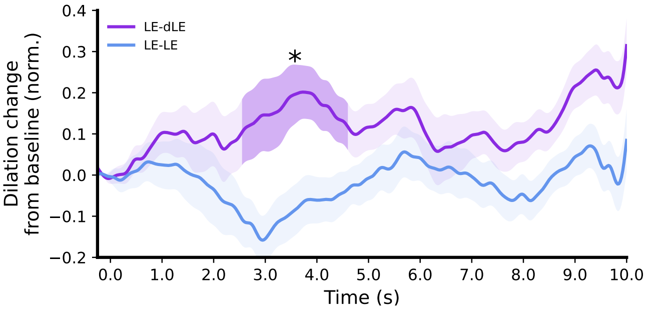

(c) HE–LE

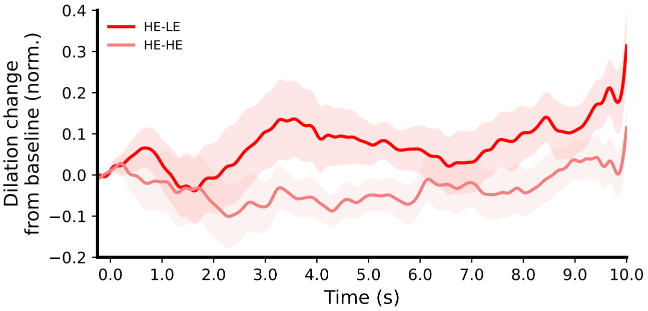

Figure S4: **Extended boundary-related pupil responses across the full post-boundary window.** Extending the analysis to the complete post-boundary segment (*40 tones*, corresponding to *10 s*) did not meaningfully alter the results or interpretation. Event-related pupil time courses are shown for all boundary transitions without temporal cropping. Lightly shaded regions denote bootstrapped standard errors across participants, while dark shading indicates significant time windows identified using cluster-based permutation testing performed over the full 10-second window (Bonferroni-adjusted $p < 0.05$).
